# VRK1 promotes epithelial-mesenchymal transition in hepatocellular carcinoma mediated by SNAI1 via phosphorylating CHD1L

**DOI:** 10.1038/s41419-025-07641-w

**Published:** 2025-04-15

**Authors:** Jing Li, Zan Song, Xue Dong, Leilei Li, Xinyu Gu, Kailing Zhang, Zhicheng Zhang, Yu Li, Zhili Fan, Hao Dong, Ying Liu, Mengfei Liu, Huiqing Zhang, Wu Liu, Tao Zhang

**Affiliations:** 1 Institute of Immunopharmaceutical Sciences, State Key Laboratory of Discovery and Utilization of Functional Components in Traditional Chinese Medicine, NMPA Key Laboratory for Technology Research and Evaluation of Drug Products, Key Laboratory of Chemical Biology, School of Pharmaceutical Sciences, Cheeloo College of Medicine, Shandong University, Jinan, Shandong China; 2 The Department of Gastrointestinal Medical Oncology, JXHC Key Laboratory of Tumor Microenvironment and Immunoregulation, Jiangxi Cancer Hospital, Nanchang, Jiangxi China; 3 State Key Laboratory for Chemistry and Molecular Engineering of Medicinal Resources, Guangxi Normal University, Guilin, Guangxi China

**Keywords:** Oncogenes, Metastasis

## Abstract

Vaccinia-related kinase 1 (VRK1) is involved in numerous cellular processes, including DNA repair, cell cycle and cell proliferation. However, its roles and molecular mechanism underlying the progression of hepatocellular carcinoma (HCC) are yet largely unexplored. Here, we demonstrated that VRK1 expression is elevated in HCC tumor tissues, which is associated with high tumor stage and poor prognosis in HCC patients. In vitro and in vivo experiments manifested that VRK1 overexpression significantly promotes cell proliferation, colony formation, migration and tumor growth of HCC by inducing epithelial-mesenchymal transition (EMT) program. Mechanistically, immunoprecipitation combined with mass spectrometry analysis determined that VRK1 interacts with CHD1L, which mediates the phosphorylation of CHD1L at serine 122 site. RNA-seq revealed that one of the key downstream target genes of VRK1 is *SNAI1*, by which VRK1 promotes EMT process and HCC progression. Furthermore, VRK1 upregulates SNAI1 expression through phosphorylating CHD1L. In conclusion, these findings suggested that VRK1/CHD1L/SNAI1 axis acts as a cancer-driving pathway to promote the proliferation and EMT of HCC, indicating that targeting VRK1 may be an attractive therapeutic strategy of HCC.

## Introduction

Liver cancer ranks the sixth in malignant cancer incidence and is the fourth in mortality rate among most commonly diagnosed cancers worldwide in 2020 [1, 2]. Hepatocellular carcinoma (HCC) accounts for ~90% of primary liver cancer cases, in which alcohol and chronic infections by hepatitis B (HBV) and C virus (HCV) are the most prominent risk factors [3]. The systemic therapies have been approved for advanced HCC, such as tyrosine kinase inhibitor (TKI, sorafenib and lenvatinib) and combinations of immunotherapies with vascular endothelial growth factor (VEGF)A monoclonal antibodies [4]. However, HCC metastasis and drug resistance remarkably result in worse prognosis and shorter survival. Therefore, it is urgently need to unveil the molecular mechanisms and new therapeutic targets in HCC progression.

Vaccinia-related kinase 1 (VRK1) predominantly localizes in the nucleus, which serves as a serine/threonine (Ser/Thr) protein kinase [5]. The VRK1 kinase directly phosphorylates several nuclear substrates to participate in multiple cellular functions including cell mitosis and proliferation, migration and DNA damage responses [6, 7]. As an oncogenic driver, VRK1 expression is highly increased in several types of cancer, such as breast cancer, ovarian cancer, non-small cell lung cancer, head and neck squamous cell carcinomas, glioma and neuroblastomas [7–9]. It was reported that enhanced VRK1 translation upregulates CCND1 expression by phosphorylating CREB, thus promoting cell proliferation and cell cycle progression in lung cancer cells [10]. Notably, VRK1 is involved in the maintenance of genomic stability and prevents cellular damages from ionizing radiation and chemotherapy agents including olaparib, doxorubicin and cisplatin [6, 11, 12]. In HCC, VRK1 modulates G1/S cell cycle transition, cell proliferation and is associated with tumor immune infiltration anti-PD-L1 immunotherapy response [13–16]. However, the role and mechanisms by which VRK1 promotes epithelial-mesenchymal transition (EMT) and HCC progression have not yet been elucidated.

In this study, we demonstrated that *SNAI1* is a downstream target of VRK1. The transcription factor SNAI1 promotes the EMT process, which inhibits the expression of the epithelial marker E-cadherin and enhances cell motility and invasiveness [17, 18]. Mounting evidence showed that SNAI1 expression is modulated at the transcriptional level, translational level and post-translational modifications [19–21]. Nonetheless, it is unclear about the underlying mechanism by which VRK1 regulates SNAI1 expression.

Importantly, we identified that VRK1 promotes SNAI1 expression by interacting with a novel substrate chromodomain helicase DNA binding protein 1-like (CHD1L). VRK1 phosphorylates CHD1L at serine 122 to induce the expression of SNAI1, thereby promoting the proliferation, migration and tumor growth of liver cancer cells. Thus, VRK1 may serve as a promising therapeutic target in liver cancer.

## Materials and methods

### Cell culture and transfection

Huh7, HepG2, Hep3B, SK-HEP-1, PLC/PRF/5 and HEK 293T cell lines were acquired from the Cell Bank of the Chinese Academy of Sciences (Shanghai, China). THLE-2 cell line was obtained from the American Type Culture Collection (ATCC, USA). Huh7, HepG2 and HEK 293T cells were maintained in Dulbecco’s modified eagle medium (DMEM, Gibco) supplemented with 10% fetal bovine serum (FBS, ExCell Bio, China) and 1% Penicillin/Streptomycin solution (Beyotime, China). Hep3B, SK-HEP-1, PLC/PRF/5 cells were cultured in Minimum Essential Media (MEM, Gibco) supplemented with 10% FBS (ExCell Bio) and 1% Penicillin/Streptomycin solution (Beyotime). THLE-2 cell was maintained in Bronchial Epithelial Cell Growth Medium (CC3170, BEGM, Lonza Bioscience, USA) supplemented with extra 5 ng/mL EGF (PeproTech, USA), 70 ng/mL Phosphoethanolamine (Sigma-Aldrich, USA) and 10% FBS (ExCell Bio). All cells were authenticated by short tandem repeat (STR) profiling and incubated at 37 °C in an atmosphere of 5% CO_2_. The transient overexpression plasmids were transfected into cells using Polyethylenimine (PEI) (24765, Polysciences, USA) at 60%–70% confluence in accordance with the manufacturer’s instructions.

### Plasmid construction

The shRNA plasmids (sh*VRK1* sequences: 5’-CCTGGTGTTGAAGATACGGAA-3’; 5’-GTAGATTATGGCCTTGCTTAT-3’; and 5’-AGATAATAACTGACATGGCAA-3’) were used to knockdown VRK1 expression from Genechem (Shanghai, China). The *SNAI1* sgRNA (5’-CACCGGTCGCCTGCATATGTTACAC-3’) was introduced into Lenti-CRISPRV2-GFP vector. To generate the CHD1L overexpression plasmid, the human full-length coding sequence (CDS) of *CHD1L* was amplified by PCR using a template generously provided by Dr. Ningfang Ma (Guangzhou Medical University, China) and subcloned into pcDNA3.1-3×HA-N (Miaoling Biology, China) at the *Kpn* I and *BamH* I sites. *CHD1L* S122 site plasmid mutation was generated using the fast mutagenesis system kit (FM111-01, TransGen Biotech, China) according to the manufacturer’s instructions.

### Lentiviral transduction and stable cell line development

To generate Huh7 and HepG2 cell lines with stable knockdown of *VRK1*, *VRK1* shRNA plasmids were co-transfected with psPAX2 and pMD2.G into HEK 293T using PEI reagent. To maximize knockdown efficiency, we used a pool of three shRNAs targeting different regions of *VRK1* for simultaneous infection. Lentiviruses were collected at 48 h after the medium change and then infected Huh7 or HepG2 cells for 48 h in the presence of 8 μg/mL polybrene (Millipore, USA). The infected cells were selected with 2 μg/mL puromycin (ST551, Beyotime) for 1 week and then evaluated the knockdown efficacy. For generation of SNAI1 knockout cell line, SNAI1 sgRNA plasmid was co-transfected with psPAX2 and pMD2.G into HEK 293T using PEI reagent. Lentiviruses were collected and then infected VRK1 knockdown Huh7 cells for 48 h with 8 μg/mL polybrene (Millipore). The GFP-positive cells were selected by flow cytometry and then evaluated the knockdown efficacy.

### Cell counting kit-8 (CCK8) assay

For cell viability assay, Huh7 cells were seeded in 96-well plates at 4000 cells per well (HepG2 cells at 5000 cells per well) with three replicates. The cells were cultured at 24 h, 48 h, 72 h, and 96 h after seeding and incubated with 10 μL CCK8 (A311, Vazyme, China) for 3 h. The absorbance was measured using a microplate reader (Bio-Tek, USA) at 450 nm.

### EdU assay

Cells were seeded into 96-well plates at a density of 8000 cells each well with three replicates. After culturing for 24 h, the cell proliferation ability was detected by the EdU assay kit (C0075S, Beyotime) according to the manufacturer’s instructions. In brief, cells were incubated with 100 μL 50 μM EdU buffer at 37 °C, 5% CO2 for 2 h, fixed with 4% polyformaldehyde for 15 min and permeabilized with 0.3% Triton X-100 for 15 min. EdU staining was performed with the reaction buffer for 30 min followed by staining of nuclei with Hoechst 33342 for 15 min and then the images were captured by a fluorescence microscope.

### Colony formation assay

Huh7 cells were seeded in 12-well plates at 2000 cells per well (HepG2 cells at 1000 cells per well) with three replicates and were cultured for about 14 days. Then the cells were washed twice with PBS, fixed with methanol for 30 min, and stained with 0.1% crystal violet solution for 30 min.

### Cell migration assay

The 24-well Plate with 8 µm transparent PET membrane (353097, Corning, USA) was used to determine cell migration capability. Huh7 cells were seeded in the upper chamber at 40,000 cells per well (HepG2 cells at 50,000 cells per well) with serum-free medium. A total of 500 μL medium with 10% FBS was added to the lower chamber. After 24 h incubation, the cells were washed with PBS, fixed with methanol for 30 min, and stained with 0.1% crystal violet solution for 30 min. The residual crystal violet solution was removed and then the images were obtained using a microscope.

### Western blotting and immunohistochemistry (IHC)

Protein was extracted using RIPA lysis solution (P0013K, Beyotime) with protease inhibitors (B14002, Bimake, China) and phosphatase inhibitors (B15001, Bimake), separated by 10% SDS-PAGE and then transferred to 0.45 μm PVDF membranes (10600023, Cytiva, USA). The PVDF membranes were blocked using 5% skimmed milk for 2 h and incubated using primary antibodies at 4 °C overnight. Anti-VRK1 antibody (A7745), anti-CHD1L antibody (A17558), anti-pan-Phospho-Ser/Thr antibody (AP1067) and anti-β-actin antibody (AC026) were purchased from ABclonal (Wuhan, China). CHD1L rabbit monoclonal antibody (R383038) was obtained from Zenbio (Chengdu, China). Anti- SNAI1 antibody (3879), anti-HA-Tag antibody (3724S) and anti-Flag-Tag antibody (14793S) were purchased from Cell Signaling Technology (CST, MA, USA). Anti-E-cadherin antibody (20874-1-AP), anti-N-cadherin antibody (22018-1-AP), anti-Vimentin antibody (10366-1-AP), anti-GAPDH antibody (60004-1-Ig) were purchased from Proteintech (Wuhan, China). On the following day, the PVDF membranes were washed by TBST 5 times and then incubated by the secondary antibodies for 2 h at room temperature. Anti-Rabbit IgG antibody (7074) and Anti-Mouse IgG antibody (AS003) were purchased from CST and ABclonal, respectively. The protein bands were visualized using an ultrasensitive ECL chemiluminescent detection kit (PK10003, Proteintech) and an imaging analysis system (Tanon, China).

The tissue microarray was obtained from Outdo Biotech Company (Shanghai, China) and IHC was carried out according to the standard protocol. Briefly, the slide was deparaffinized, rehydrated and performed antigen retrieval. Then, the tissue was incubated with anti-VRK1 antibody at 4 °C overnight and secondary antibody for 45 min, and developed with DAB staining. QuPath, an open-source image analysis software available at https://qupath.github.io was used for tissue microarrays analysis. The detached tissues were excluded for analysis.

### Co-immunoprecipitation (Co-IP) and mass spectrometry

The appropriate amount of anti-Flag magnetic beads (B26102, Bimake) or anti-HA magnetic beads (B26202, Bimake) were pretreated using TBS according to the manufacturer’s instructions. Protein A/G magnetic beads were obtained and used for immunoprecipitation of endogenous CHD1L protein (88802, Thermo Scientific, USA). The protein was extracted using IP lysis solution (P0013J, Beyotime) with protease and phosphatase inhibitors and then incubated with the pretreated magnetic beads overnight at 4 °C. Protein-bound magnetic beads were washed with PBST 5 times at 2 min per time and then the protein was eluted using protein sample loading buffer (P1016, Solarbio, China) and boiled at 98 °C for 10 min.

The proteins were immunoprecipitated using magnetic beads and then separated using SDS-PAGE. The gel was stained using coomassie blue superfast staining solution (P0017F, Beyotime). The excised gels were digested and liquid chromatography tandem mass spectrometry analysis was performed using a Q-Exactive mass spectrometer coupled with an Easy nLC (Thermo Scientific) from Applied Protein Technology (APTBIO, Shanghai, China).

### RNA sequencing

Total RNA from control cells and VRK1 knockdown cells was extracted with RNAprep pure cell kit (DP430, TIANGEN, China). The RNA quality was confirmed using Fragment Analyzer (Agilent, USA) and then RNA sequencing was performed using the DNBSEQ platform from BGI Genomics (Wuhan, China). The differentially expressed genes were identified by DESeq2 analysis. The Kyoto Encyclopedia of Genes and Genomes (KEGG) and Gene Set Enrichment Analysis (GSEA) were performed using Dr. Tom online system.

### Real-time PCR (qRT-PCR)

Total RNA was extracted with an RNAfast200 kit (Fastagen, China). cDNA was synthesized using ReverTra Ace qPCR RT Master Mix with gDNA Remover (FSQ-301, TOYOBO, Japan). qRT-PCR was conducted using SYBR Green (Q311,Vazyme) operated in the LightCycler 480II Real-time PCR system (Roche, Switzerland). The relative RNA level was calculated by the comparative C_T_ method with the normalization to *GAPDH*. The PCR primers were listed in Supplementary Table 1.

### Tumor xenograft in nude mice

A total of 4 × 10^6^ Huh7 cells from control or VRK1 knockdown groups were suspended in 140 μL PBS and injected subcutaneously into 5-week-old male BALB/c nude mice obtained from Beijing Vitalstar Biotechnology. During the process of tumor formation, the weight of the nude mice and the tumor length (L) and width (W) were measured daily. Tumor volumes (mm^3^) were calculated using the formula: L × (W)^2^/2. When the tumor volume reached 1500 mm^3^, the nude mice were sacrificed. The tumor tissues were separated for subsequent experiments. All mice were fed under specific pathogen-free conditions at the model animal research center of Shandong University (Jinan, Shandong, China) under a regular 12-h light/dark schedule at a constant room temperature (20°C to 24 °C). All animal experiments were approved by the Animal Care and Use Committee of Shandong University.

### Statistical analysis

The experimental data were statistically analyzed using GraphPad Prism 8.0.1 software. The results are shown as mean ± standard deviation (mean ± SD). Student’s *t* test was used to compare two groups. One-way ANOVA followed by Tukey test was used to compare multiple groups. Statistical significance was represented as *p < 0.05, **p < 0.01, ***p < 0.001 and ****p < 0.0001.

## Results

### VRK1 is highly expressed and associated with poor prognosis in HCC

**Fig. 1:**
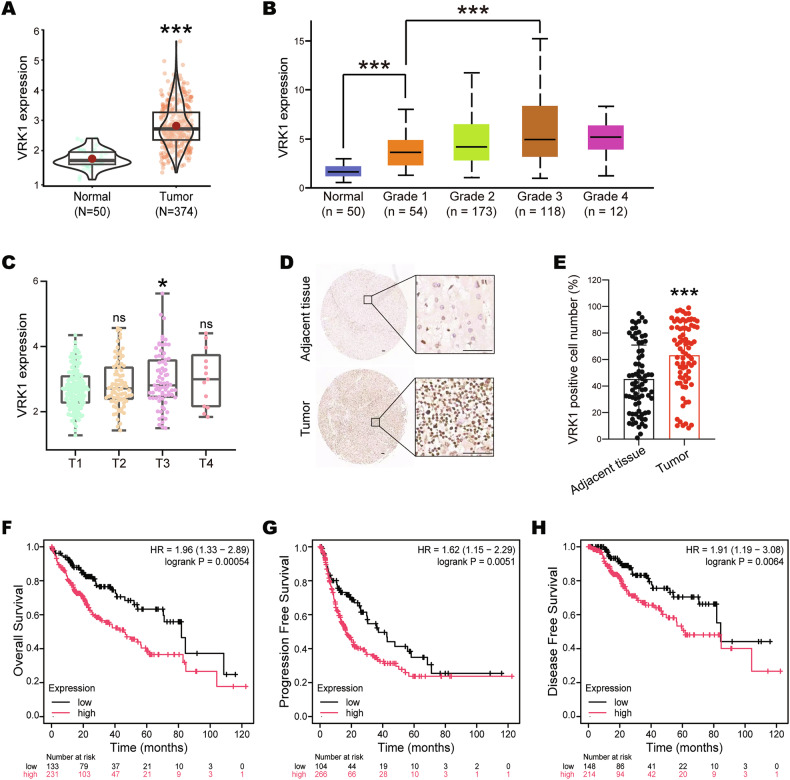
High VRK1 expression is associated with poor prognosis in liver cancer. **A** VRK1 expression in normal tissues and liver tumor tissues (compared with normal tissue, ***p < 0.001). **B** Relationship between VRK1 expression and tumor grade of liver cancer patients (data analysis website: https://ualcan.path.uab.edu/; Grade 1: well-differentiated; Grade 2: moderately differentiated; Grade 3: poorly differentiated; Grade 4: undifferentiated; Normal vs. Grade 1, ***p < 0.001; Grade 1 vs. Grade 3, ***p < 0.001). **C** Relationship between VRK1 expression and tumor stage in liver cancer patients (compared with T1 stage, *p < 0.05). Representative images (**D**) and IHC score (**E**) of VRK1 staining in adjacent and HCC tumor tissues (compared with adjacent tissue, ***p < 0.001). Correlation between VRK1 expression and overall survival (**F**), progression free survival (**G**), disease free survival (**H**).

VRK1 mRNA levels were examined in HCC tissues by analyses of the Cancer Genome Atlas (TCGA) data. The results showed that VRK1 was significantly overexpressed in HCC tissues compared with the non-tumor liver tissues (Fig. 1A). Moreover, we found that VRK1 expression was upregulated in the tumor grade 3 and cancer stage T3 (Fig. 1B, C). In addition, the immunohistochemistry (IHC) staining on HCC tissue microarray was performed. The data revealed that VRK1 showed a strong nuclear staining and demonstrated a significant increase in HCC tumor tissues compared with the adjacent non-tumor tissues (Fig. 1D, E). More importantly, further Kaplan-Meier survival analyses indicated that the HCC patients with high VRK1 expression exhibited much shorter overall survival, progression free survival and disease-free survival (Fig. 1F–H), suggesting that VRK1 is highly expressed and correlated with much worse prognosis in HCC.

### Depletion of VRK1 inhibits cell proliferation and migration in HCC

**Fig. 2:**
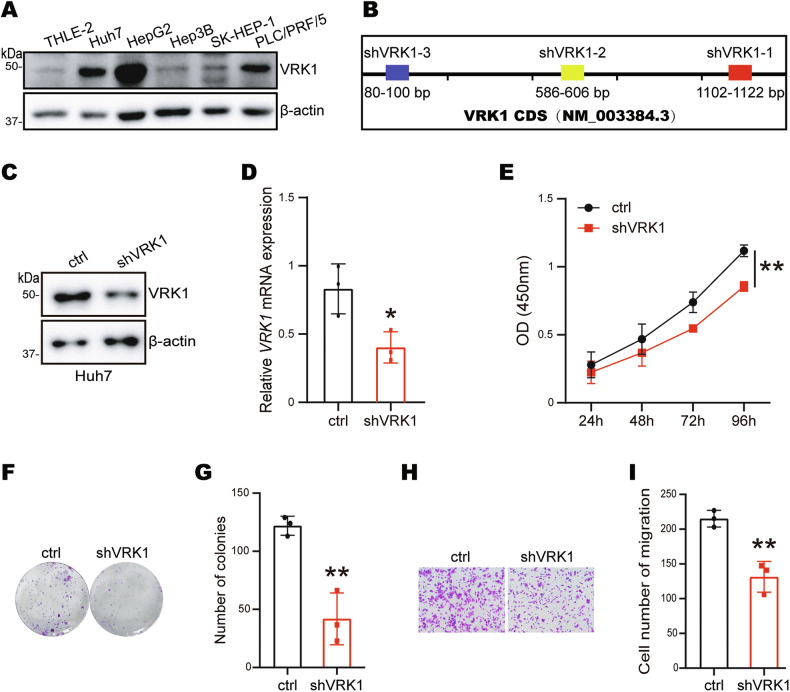
VRK1 promotes the proliferation and migration of liver cancer cells. **A** The expression of VRK1 in normal liver cells and liver cancer cells. **B** Diagram of VRK1 shRNA targeting region in VRK1 coding sequence. Immunoblots analysis of VRK1 protein level (**C**) and qRT-PCR analysis of VRK1 mRNA level (**D**) in Huh7 cells infected with VRK1 shRNA. CCK8 (**E**), colony formation (**F**, **G**) and transwell assay (**H**, **I**) were determined in Huh7 cells. Data are represented as means ± SD relative to the control group (*n* = 3). *p < 0.05, **p < 0.01.

We next detected VRK1 protein expression levels in normal hepatocytes and several liver cancer cell lines. Intriguingly, VRK1 protein levels were sharply upregulated in liver cancer cell lines Huh7 and HepG2 cells compared with normal hepatocytes THLE-2 (Fig. 2A). To further investigate the effect of VRK1 on the function of HCC cells, we selected Huh7 and HepG2 cells to generate stable knockdown of VRK1 cell lines using three different shRNA targeting coding sequence of *VRK1* gene (Fig. 2B). We observed that the VRK1 mRNA and protein expression levels were significantly reduced in Huh7 (Fig. 2C, D) and HepG2 cells upon ablation of VRK1 (Supplementary Fig. 1A, B). Interestingly, knockdown of VRK1 decreased the cell viability in Huh7 (Fig. 2E) and HepG2 cells (Supplementary Fig. 1C). Similarly, depletion of VRK1 also reduced cell colonies number and size in Huh7 cells (Fig. 2F, G) but not in HepG2 cells (Supplementary Fig. 1D, E), suggesting that VRK1 promotes colony formation in HCC cells in a context-dependent manner. Then, we performed Transwell assay to evaluate the effect of VRK1 on cell migration capacity. The results showed that ablation of VRK1 decreased cell migration number in Huh7 (Fig. 2H, I) and HepG2 cells (Supplementary Fig. 1F, G). Moreover, we demonstrated that transient VRK1 expression increased colony formation and the number of migrated cells (Supplementary Fig. 1H, I). Taken together, VRK1 enhances the proliferation and migration of hepatocellular carcinoma cells.

### VRK1 interacts with and phosphorylates CHD1L on serine 122

**Fig. 3:**
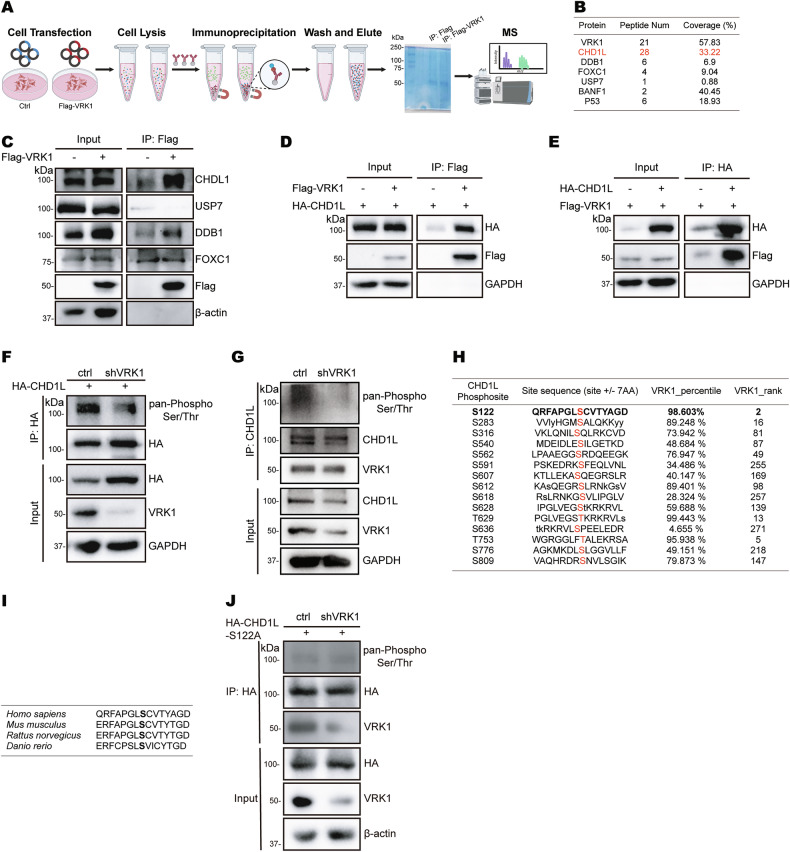
VRK1 interacts with CHD1L and phosphorylates the CHD1L S122 site. **A** Schematic diagram of the screening approach of VRK1 interacting protein by immunoprecipitation combined with mass spectrometry analysis. **B** Mass spectral peptide count and coverage (%) of VRK1-interacting proteins. Peptide Num number of peptides used for characterization, Coverage (%) peptide coverage. **C** Huh7 cells were introduced with the control plasmid and transient overexpression of Flag-VRK1, then immunoprecipitated by Flag-VRK1 protein and confirmed its interacting proteins by Western blot. **D**, **E** Exogenous interactions between Flag-VRK1 and HA-CHD1L were detected by immunoprecipitation in Huh7 cells. **F** HA-CHD1L plasmid was transfected into Huh7 cells with silencing VRK1. The serine and threonine phosphorylation of HA-CHD1L protein immunoprecipitated by HA beads was detected by immunoblot with pan-Phospho Ser/Thr antibody. **G** The endogenous CHD1L protein was immunoprecipitated by protein A/G beads and the CHD1L phosphorylation level was determined. **H** Prediction analysis on the website (https://www.phosphosite.org) to identify CHD1L phosphorylation residue by VRK1. **I** The sequence conservation of CHD1L protein. **J** HA-CHD1L-S122A mutant was transfected into Huh7 cells with silencing VRK1. The serine and threonine phosphorylation of HA-CHD1L-S122A protein immunoprecipitated by HA beads was detected by immunoblot with pan-Phospho Ser/Thr antibody.

To decipher the regulatory mechanism by which VRK1 promotes proliferation and migration in hepatocellular carcinoma, we performed immunoprecipitation followed by mass spectrometry (IP-MS) to distinguish the potential interacting proteins of VRK1 (Fig. 3A). Barrier-to-autointegration factor (BAF) and p53, two well-known substrates of VRK1, were identified in the MS protein list, indicating that the MS results have high reliability (Fig. 3B). Moreover, co-immunoprecipitation assay was conducted to confirm whether these novel substrates CHD1L, DDB1, FOXC1 and USP7 may interact with VRK1. The results demonstrated that endogenous CHD1L could bind with VRK1 (Fig. 3B, C). CHD1L is a multifunctional protein that participates in diverse cellular processes, including chromosome remodeling, cell differentiation and DNA repair [22, 23]. *CHD1L* has recently been characterized as a driver gene, and hence plays vital roles in tumor progression and sorafenib resistance of HCC [24, 25]. To further determine the physical interaction between VRK1 and CHD1L, co-immunoprecipitation assay was performed in Huh7 cells and 293T cells with ectopic expression of Flag-VRK1 and HA-CHD1L. The results determined reciprocal interaction between VRK1 and CHD1L protein (Fig. 3D, E and Supplementary Fig. 2A, B).

VRK1 is a nuclear Ser/Thr chromatin kinase and usually promotes carcinogenesis by phosphorylating its substrates. Thus, we supposed that VRK1 may modulate CHD1L phosphorylation. To support this hypothesis, we first investigated whether VRK1 influenced global phosphorylation levels of exogenous CHD1L. Intriguingly, the results indicated that knockdown of VRK1 largely abrogated overall phosphorylation modification levels of exogenous CHD1L (Fig. 3F). Subsequently, we detected the effect of VRK1 on endogenous CHD1L levels. VRK1 depletion reduced endogenous p-CHD1L (Fig. 3G), but didn’t affect CHD1L mRNA and protein expression (Supplementary Fig. 2C). To explore phosphorylated amino acid residues of CHD1L by VRK1 kinase, we predicted potential phosphorylation sites using an online phosphositeplus website (https://www.phosphosite.org). The results showed that CHD1L was possibly phosphorylated on the serine 122 by VRK1 (Fig. 3H). In addition, we analyzed sequence conservation of CHD1L protein from different organisms. Interestingly, the serine 122 was a conserved amino acid of CHD1L protein across many species (Fig. 3I). Therefore, we introduced a phospho-dead mutant of CHD1L by substituting serine with alanine (S122A) and detected the effect of VRK1 on its phosphorylation. We found that knockdown of VRK1 didn’t reduce the phosphorylation level of CHD1L S122A (Fig. 3J), suggesting that VRK1 phosphorylates CHD1L at the evolutionally conserved Ser122 residue.

### *SNAI1* is a key downstream target gene of VRK1

**Fig. 4:**
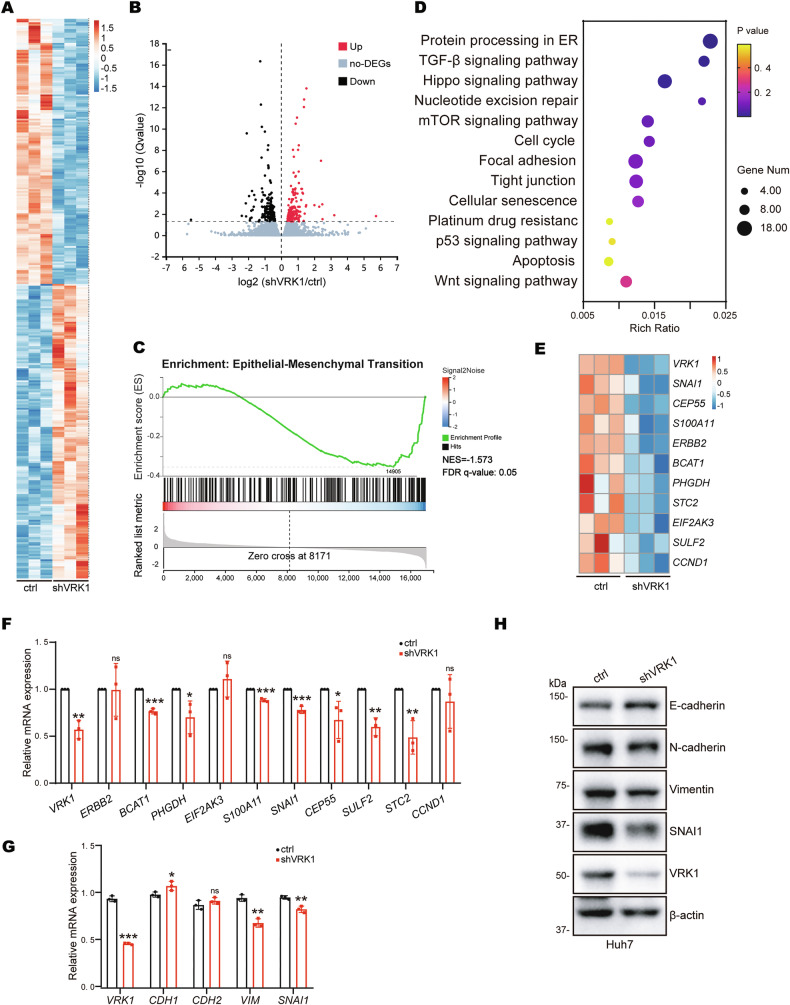
*SNAI1* is a key downstream target gene of VRK1. The heatmap (**A**) and the volcano plot (**B**) of RNA-seq of the control group and VRK1 knockdown group in Huh7 cells (*n* = 3). GSEA analysis (**C**), KEGG pathway enrichment analysis (**D**), heatmap analysis (**E**) of the top significantly differentially expressed genes between the control group and VRK1 knockdown group in Huh7 cells. **F** qRT-PCR analysis of mRNA level of the top significantly differentially expressed genes. The EMT-related genes expression was detected by qRT-PCR (**G**) and immunoblots (**H**).

To dissect the downstream target genes regulated by VRK1, we performed RNA sequencing analyses in Huh7 cells with stable VRK1 knockdown and the corresponding control cells. We found that a total of 325 differentially expressed genes were identified, among which 154 genes were downregulated and the remaining 171 genes were upregulated upon VRK1 depletion (Fig. 4A, B). Gene Set Enrichment Analysis (GSEA) indicated that the epithelial-mesenchymal transition (EMT) related gene signature was positively enriched in VRK1 knockdown cells compared to control cells (Fig. 4C). Moreover, the Kyoto Encyclopedia of Genes and Genomes (KEGG) pathway analyses confirmed that VRK1 was closely associated with crucial EMT-associated processes, such as focal adhesion and tight junction (Fig. 4D). Therefore, the mRNA expression of thirteen EMT-related genes was further determined by qRT-PCR. We found that depletion of VRK1 sharply inhibited the mRNA levels of *BCAT1*, *S100A11*, *CEP55* and *SNAI1*, and enhanced *CDH1* mRNA level in both Huh7 and HepG2 cells (Fig. 4E–G and Supplementary Fig. 3A, B). More significantly, we found that VRK1 knockdown reduced the protein expression of mesenchymal cell marker SNAI1 and induced the expression of epithelial cell marker E-cadherin (Fig. 4H and Supplementary Fig. 3C). Collectively, VRK1 is engaged in the EMT processes by modulating SNAI1 in HCC.

### VRK1 promotes proliferation and migration partly through CHD1L/SNAI1 in HCC

**Fig. 5:**
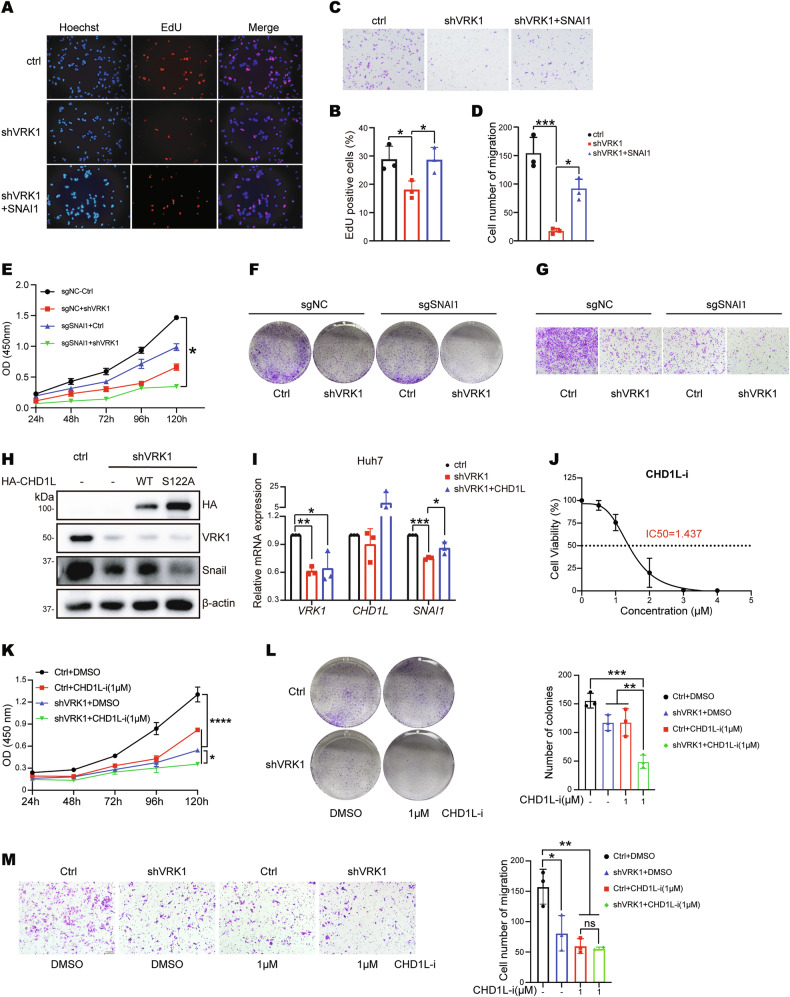
VRK1 promotes liver cancer progression partly through CHD1L/SNAI1. EdU (**A**, **B**) and transwell assay (**C**, **D**) were performed in Huh7 cells with VRK1 knockdown after rescue with SNAI1 plasmid. Data are represented as means ± SD (*n* = 3). shVRK1 vs. ctrl, ***p < 0.001, *p < 0.05; shVRK1 + SNAI1 vs. shVRK1, *p < 0.05. CCK8 assay (**E**), colony formation (**F**) and transwell assay (**G**) were performed using either control or VRK1 knockdown cells under depletion of SNAI1. *p < 0.05. **H** The protein level of SNAI1 was detected in the control group, VRK1 knockdown group, VRK1 knockdown and overexpressing HA-CHD1L group, and VRK1 knockdown and overexpressing HA-CHD1L-S122A mutant group of Huh7 cells by Western blot, respectively. **I** The mRNA level of SNAI1 was detected by qRT-PCR in the control group, VRK1 knockdown group, VRK1 knockdown and overexpressing HA-CHD1L group of Huh7 cells, respectively. Data are represented as means ± SD (*n* = 3). shVRK1 vs. ctrl, **p < 0.01, *p < 0.05; shVRK1 + CHD1L vs. ctrl, *p < 0.05; shVRK1 + SNAI1 vs. shVRK1, *p < 0.05. **J** IC50 curve of CHD1L inhibitor CHD1Li6.11 in Huh7 Cells. The cell viability was detected using CCK8 assay after treatment with CHD1Li for 72 h. The IC50 value was calculated using nonlinear regression. **K**–**M** Control and VRK1 knockdown cells were treated with CHD1L inhibitor CHD1Li at 1 μM (E1146, Selleck). CCK8 assay (**K**), colony formation (**L**) and transwell assay (**M**) were conducted. *p < 0.05, **p < 0.01, ***p < 0.001 and ****p < 0.0001.

We next investigated whether VRK1 enhanced cell proliferation and migration by regulating SNAI1 expression. The EdU and transwell migration assay exhibited that depletion of VRK1 led to reduced cell proliferation and migration in Huh7 cells, while overexpression of SNAI1 partially reversed the growth inhibition and the decrease of migration induced by VRK1 knockdown in Huh7 cells (Fig. 5A–D and Supplementary Fig. 4A). Moreover, we demonstrated that VRK1 knockdown still inhibited cell proliferation, colony formation and cell migration under depletion of SNAI1 (Fig. 5E–G and Supplementary Fig. 4B). We further explored whether VRK1 regulated SNAI1 expression through phosphorylation modification of CHD1L. The results showed that knockdown of VRK1 inhibited SNAI1 expression, while this inhibition can be rescued by overexpression of wide-type CHD1L, but not phospho-dead CHD1L S122A mutant (Fig. 5H, I). In addition, CHD1Li6.11 was used to investigate the effect on cellular function, which was a selective inhibitor of CHD1L [26]. We determined that the IC50 value of CHD1Li6.11 (CHD1L-i) was 1.437 μM (Fig. 5J). Then, we further investigated the effect of CHD1L-i treatment on cellular functions in control and VRK1-knockdown Huh7 cells. The data showed that the CHD1L inhibitor alone reduced cell viability, colony formation and cell migration, while VRK1 knockdown remained this inhibitory effect under CHD1Li6.11 treatment (Fig. 5K–M). Taken together, these results indicated that VRK1 promotes cell proliferation and migration partly through CHD1L/SNAI1.

### VRK1 promotes tumor growth in vivo

**Fig. 6:**
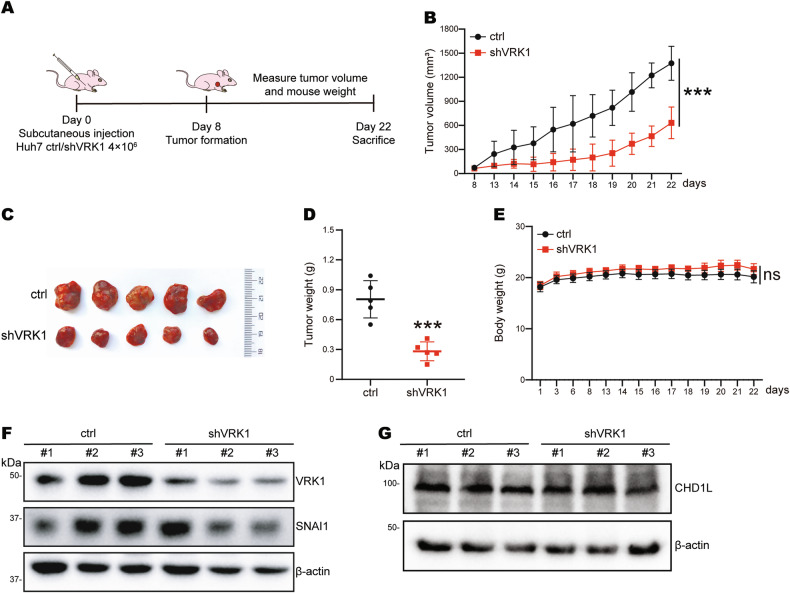
Depletion of VRK1 inhibits tumor growth in vivo. **A** Diagram of subcutaneous xenograft tumor model. Growth curve (**B**), dissected tumor images (**C**), tumor weight (**D**) and mice body weight (**E**) for the xenograft experiments with indicated cells inoculated subcutaneously into nude mice. Visible tumors were measured every day. Data are represented as means ± SD relative to the control group (*n* = 5). ***p < 0.001. The protein expression of VRK1, SNAI1 (**F**) and CHD1L (**G**) in xenograft tumor tissues.

The above-mentioned results demonstrated that VRK1 significantly enhanced cell proliferation, colony formation, and migration capacity of hepatocellular carcinoma cells in vitro. To further investigate the effects of VRK1 on tumor formation and growth in vivo, we constructed a xenograft tumor model in BALB/c nude mice. An equal number of Huh7 cells with control or VRK1 knockdown were subcutaneously injected into nude mice. After injection, the mice were closely monitored daily for body weight and tumor volume for about 2 weeks (Fig. 6A). The tumor growth rate of mice in the VRK1 knockdown group was noticeably slower than that of the control group (Fig. 6B). Consistent with this result, depletion of VRK1 significantly blocked the xenograft tumor volumes and weight (Fig. 6C, D). However, the body weight of the mice did not fluctuate significantly (Fig. 6E). In addition, we detected the protein expression levels of VRK1 and SNAI1 from mice tumor tissues. Remarkably, the protein expression of VRK1 also exhibited a positive correlation with SNAI1 expression in mice tumor tissues (Fig. 6F). However, VRK1 didn’t impact CHD1L protein levels in tumor tissues (Fig. 6G), which was consistent with the result shown in Supplementary Fig. 2C. Collectively, VRK1 regulates SNAI1 protein expression and promotes tumor growth in vivo.

## Conclusion

The protein kinases play critical roles in tumorigenesis and progression of various types of cancer [27]. Approximately 155 kinases, 30% of the human kinome, have already been recognized as potential drug targets [28]. In the present study, we identified a promising therapeutical target and explored the correlation between chromatin kinase VRK1 and clinicopathological features in HCC. By analyzing the TCGA database, we showed that VRK1 expression was significantly higher in liver tumor tissues compared with normal liver tissues. Consistently, IHC staining of VRK1 exhibited an extreme increase in HCC compared with paratumor tissues. Furthermore, VRK1 expression was upregulated in HCC patients corresponding to high tumor grade and cancer stage, indicating that VRK1 expression is positively correlated with the malignancy of liver tumor. More importantly, VRK1 expression was associated with poor prognosis in liver cancer. In vitro functional assays demonstrated that VRK1 enhanced cell proliferation, colony formation and cell migration in HCC cells. Consistent with our results, a previous study found that VRK1 expression is increased and linked to a poor prognosis in HCC patients [13]. However, the underlying mechanism by which VRK1 promotes HCC progression remains largely unexplored.

During tumor metastatic process, the tumor cells induce expression of numerous matrix metalloproteinases (MMPs) and remodel extracellular matrix (ECM) to facilitate local invasion [29]. Thousands of tumor cells further infiltrate into nearby blood vessels, while only a few cells metastasize to distant sites, and subsequently colonize in distant organs to establish tumor metastasis [29, 30]. A key event at the initiation of tumor invasion and metastasis is the epithelial-mesenchymal transition (EMT) process [18]. Recent studies confirmed that SNAI1 has been extensively characterized as a key driver of tumor aggressiveness and metastasis through contributing to the EMT program [20]. In our study, VRK1 played a vital role in modulating EMT mediated by elevated SNAI1 expression. In addition, further studies examined that VRK1 promoted the proliferation and migration of liver cancer cells by upregulating the expression of SNAI1 in vitro. Moreover, we found that VRK1 upregulated SNAI1 expression through interacting with CHD1L protein. CHD1L, a key chromatin remodeler, is involved in maintaining an open and active chromatin state to activate gene transcription [23]. Therefore, we supposed that CHD1L may promote SNAI1 transcription by chromatin remodeling, which merits further investigation. It was reported that the macro domain of CHD1L is modified by poly-ADP-ribose, which relieves the auto-inhibition status of CHD1L and mediates chromatin remodeling and PARP1 resistance [31, 32]. In HCC, CHD1L promotes tumor malignant progression and Sorafenib resistance, which is combated by PARP inhibitor olaparib [25]. Our study firstly revealed that CHD1L was phosphorylated at S122 site by VRK1. Due to high hydrophobic amino acid Cys123 and technical challenges, the specific antibody for p-CHDL1 S122 is unavailable. The p-CHDL1 level on S122 couldn’t be effectively determined in tissues. Nevertheless, whether or not CHD1L S122 phosphorylation affect clinical drug sensitivity is not well understood and worth further research.

Currently, some novel and potent VRK1 inhibitors are designed and introduced, such as dihydropteridinone derivatives, VRK-IN-1 and luteolin. Unfortunately, these dihydropteridinone derivatives also targeted CK1δ and CK1ε kinase in addition to VRK1 [33]. Interestingly, VRK-IN-1, a selective inhibitor, reduces histone H3K9 and H4K16 acetylation levels by targeting VRK1 [34]. We also determined the effect of VRK-IN-1 on cell viability in HCC and found that VRK-IN-1 did not inhibit cell proliferation at 20 μM concentration (data not shown). It was reported that luteolin, a kind of flavonoid-like natural product, suppresses the proliferation, migration and promotes cell apoptosis in multiple cancer types, including lung cancer, ovarian cancer and HCC [8, 35, 36]. However, luteolin exhibits anti-tumor activity by modulating various targets and signal pathways including VRK1, THOC1 and p53 [8, 35]. Therefore, highly selective VRK1 inhibitors or PROTAC degraders need to be developed in future.

**Fig. 7:**
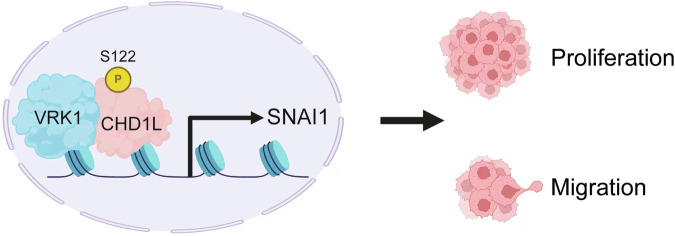
Working model of the proposed mechanism. VRK1 promotes the proliferation and migration of liver cancer cells by regulating SNAI1 expression through phosphorylation of the CHD1L S122 site.

In summary, we found that VRK1 interacted with and phosphorylated the CHD1L at S122 site to regulate SNAI1 expression, thus promoting the proliferation, migration and tumor growth of liver cancer cells (Fig. 7). This study further refines the biological functions played by VRK1 in liver cancer progression and explores the mechanism by which the VRK1-CHD1L-SNAI1 axis promotes liver cancer progression. The intervention of VRK1 may provide a promising therapeutic strategy for liver cancer.

## Supplementary information

Supplementary Figure Legend

Supplementary Figure 1

Supplementary Figure 2

Supplementary Figure 3

Supplementary Figure 4

Supplementary Table 1

Western Blot Original Data

## Data Availability

The data that support the findings of this study are available on request from the corresponding author.
