# Supplementary material for: VRK1 promotes epithelial-mesenchymal transition in hepatocellular carcinoma mediated by SNAI1 via phosphorylating CHD1L
Source: Cell Death Dis. 2025 Apr 15;16(1):302. doi: 10.1038/s41419-025-07641-w (PMC12000354; doi:10.1038/s41419-025-07641-w)
Supplement: Supplementary file 1 — Supplementary Figure Legend [file 41419_2025_7641_MOESM1_ESM.docx]

**Supplementary Figure 1**

A, B. Immunoblots analysis of VRK1 protein level (A) and qRT-PCR analysis of VRK1 mRNA level (B) in HepG2 cells infected with VRK1 shRNA.

C-G. CCK8 (C), colony formation (D, E) and Transwell assay (F, G) were determined in HepG2 cells.

H, I. Colony formation (H) and Transwell assay (I) were performed in VRK1 overexpressed Huh7 cells.

Data are represented as means ± SD relative to the control group (n = 3). ^*^*p* < 0.05, ^**^*p* < 0.01, ^***^*p* < 0.001.

**Supplementary Figure 2**

A, B. Exogenous interactions between Flag-VRK1 and HA-CHD1L were detected by immunoprecipitation in 293T cells.

C. The expression of CHD1L mRNA (left panel) and protein levels (right panel) were measured in VRK1 knockdown Huh7 and HepG2 cells.

**Supplementary Figure 3**

A. qRT-PCR analysis of mRNA level of the top significantly differentially expressed genes in HepG2 cells.

B, C. The EMT-related genes expression was detected by qRT-PCR (B) and immunoblots (C) in HepG2 cells. Data are represented as means ± SD relative to the control group (n = 3). ^**^*p* < 0.01, ^***^*p* < 0.001.

**Supplementary Figure 4**

A. Immunoblots detecting the SNAI1 expression in Huh7 cells after transfecting the SNAI1-overexpress-plasmid.

B．Western blot analysis of SNAI1 protein expression in the SNAI1 knockout Huh7 cell line.
