# Supplementary figures and images for: VRK1 promotes epithelial-mesenchymal transition in hepatocellular carcinoma mediated by SNAI1 via phosphorylating CHD1L
Source: Cell Death Dis. 2025 Apr 15;16(1):302. doi: 10.1038/s41419-025-07641-w (PMC12000354; doi:10.1038/s41419-025-07641-w)

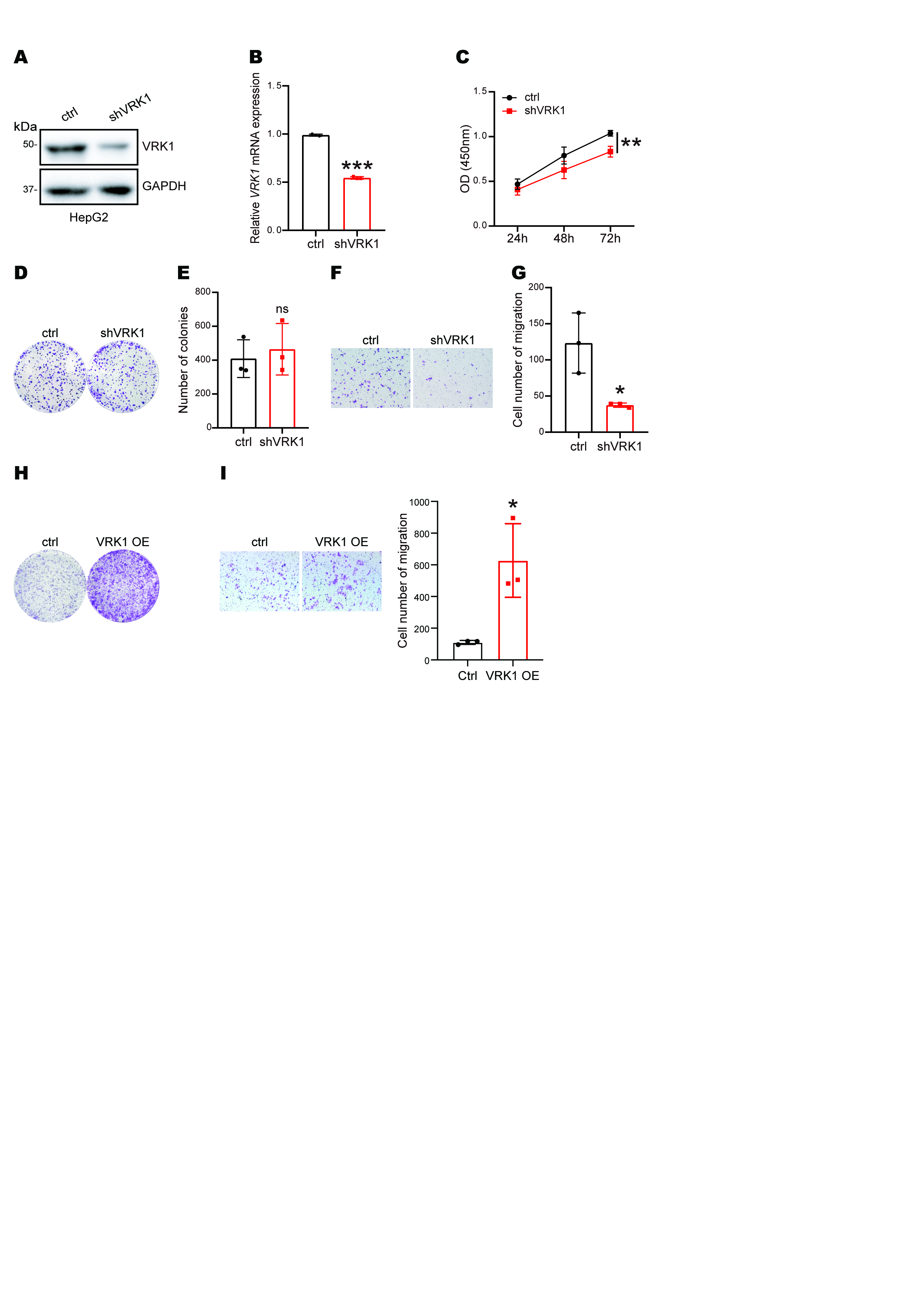

Supplement: Supplementary file 2 — Supplementary Figure 1 [file 41419_2025_7641_MOESM2_ESM.tif]

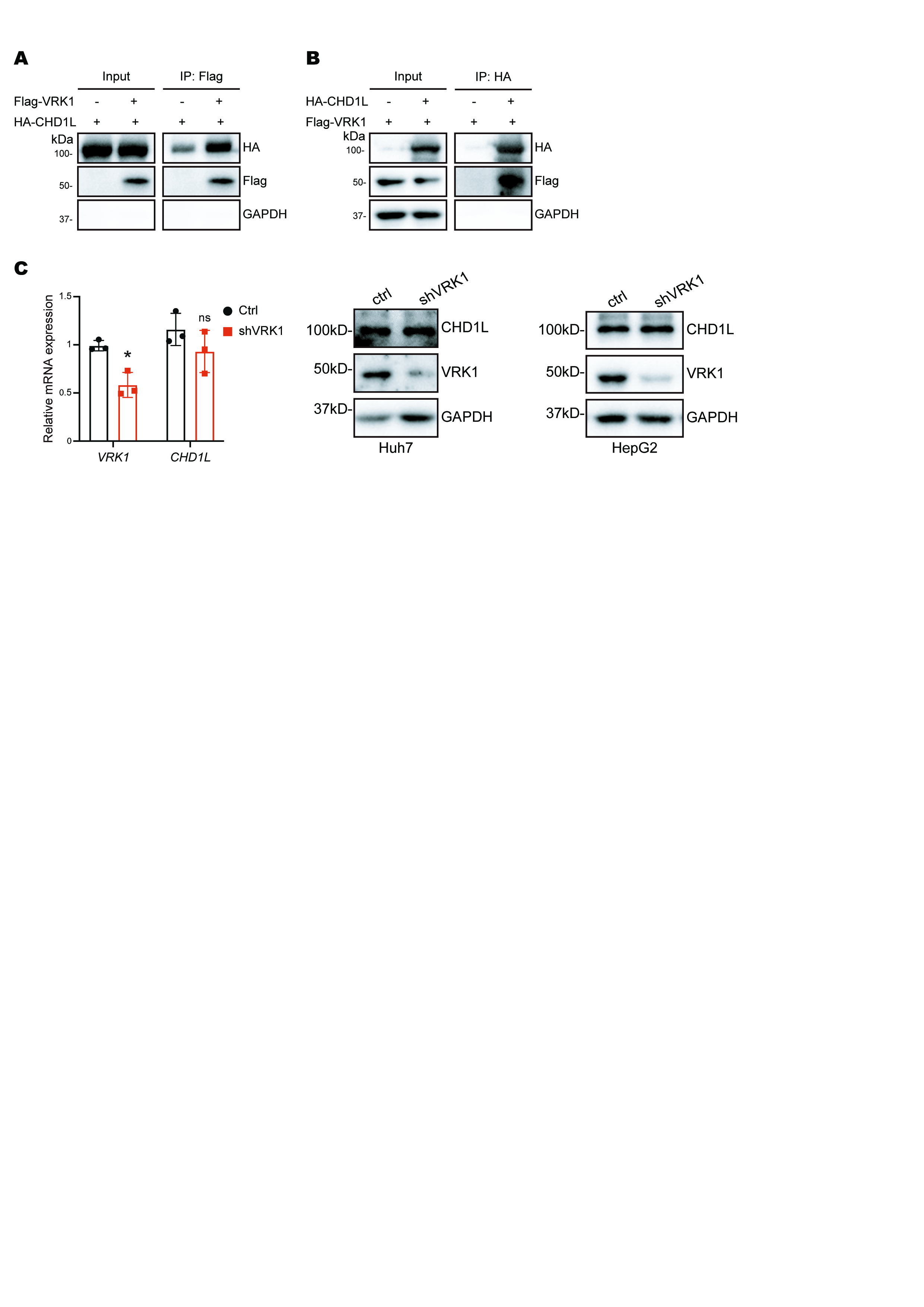

Supplement: Supplementary file 3 — Supplementary Figure 2 [file 41419_2025_7641_MOESM3_ESM.tif]

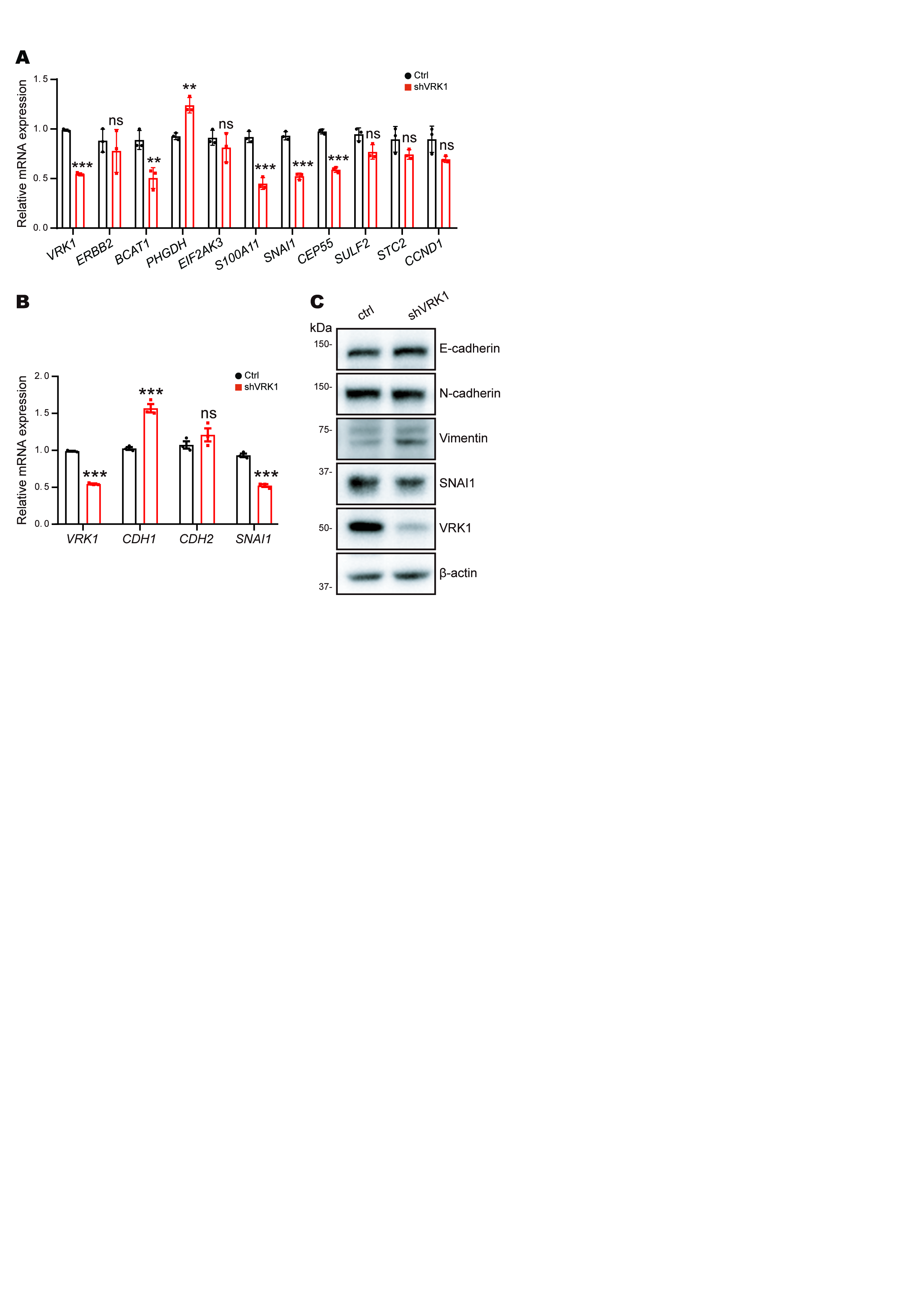

Supplement: Supplementary file 4 — Supplementary Figure 3 [file 41419_2025_7641_MOESM4_ESM.tif]

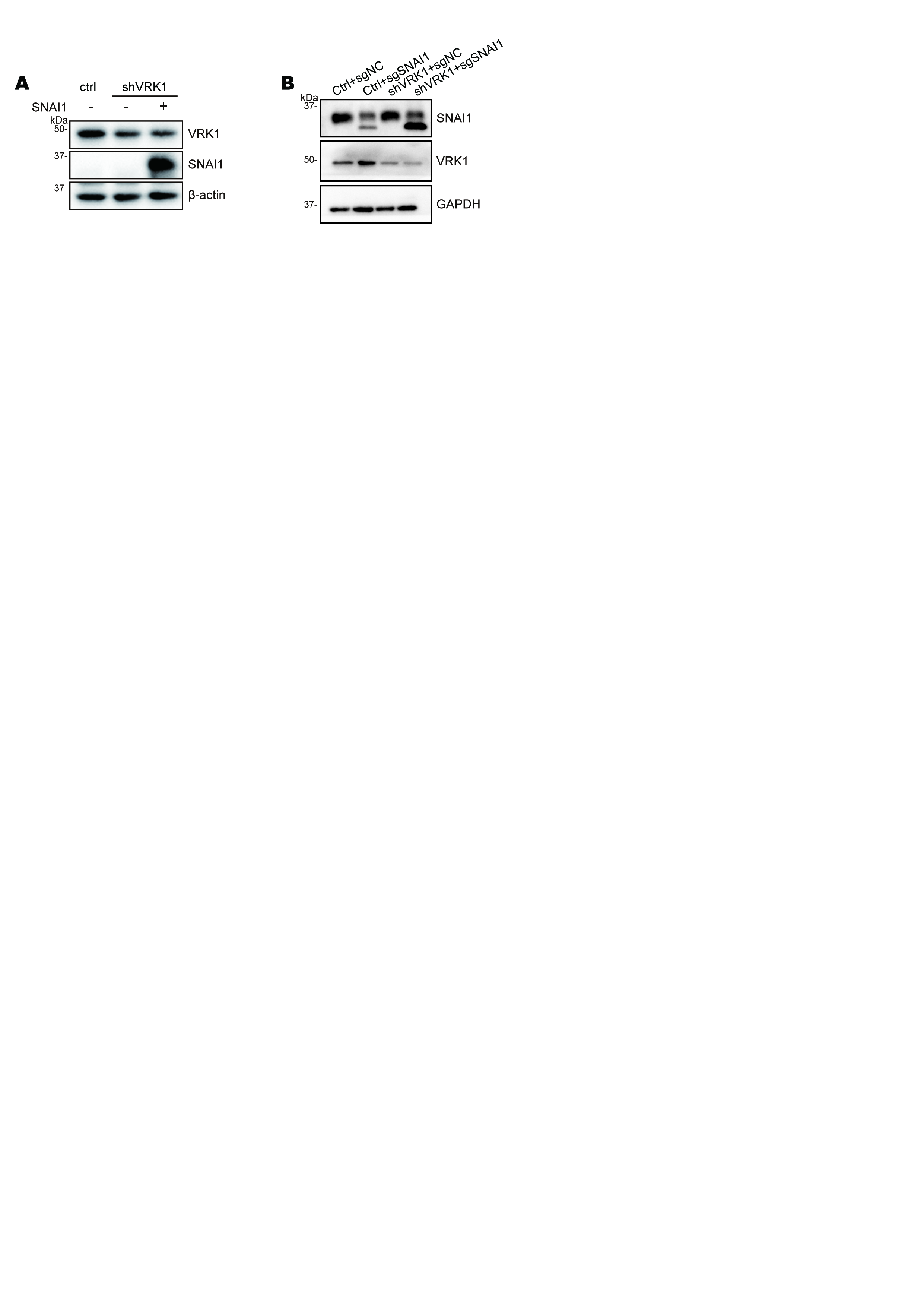

Supplement: Supplementary file 5 — Supplementary Figure 4 [file 41419_2025_7641_MOESM5_ESM.tif]
