# Supplementary material for: VRK1 promotes epithelial-mesenchymal transition in hepatocellular carcinoma mediated by SNAI1 via phosphorylating CHD1L
Source: Cell Death Dis. 2025 Apr 15;16(1):302. doi: 10.1038/s41419-025-07641-w (PMC12000354; doi:10.1038/s41419-025-07641-w)
Supplement: Supplementary file 6 — Supplementary Table 1 [file 41419_2025_7641_MOESM6_ESM.docx]

**Supplementary Table 1.** The primers of qRT-PCR

| Species | Gene | Sequences（5’→3’） |
| --- | --- | --- |
| Human | *GAPDH* | F: GGAGCGAGATCCCTCCAAAAT  R: GGCTGTTGTCATACTTCTCATGG |
| Human | *VRK1* | F: CTACCAACGAGCTGCAAAACC  R: TCACTCCCAAAGCGATCCATTA |
| Human | *CHD1L* | F: GGTGGAGTTGGCATGAACTT  R: CACTCAACTGGAGGTCAGCA |
| Human | *SNAI1* | F: TCGGAAGCCTAACTACAGCGA  R: AGATGAGCATTGGCAGCGAG |
| Human | *CDH1* | F: GCCTCCTGAAAAGAGAGTGGAAG  R: TGGCAGTGTCTCTCCAAATCCG |
| Human | *CDH2* | F: CCTCCAGAGTTTACTGCCATGAC  R: GTAGGATCTCCGCCACTGATTC |
| Human | *VIM* | F: AGGCAAAGCAGGAGTCCACTGA  R: ATCTGGCGTTCCAGGGACTCAT |
| Human | *ERBB2* | F: GGAAGTACACGATGCGGAGACT  R: ACCTTCCTCAGCTCCGTCTCTT |
| Human | *BCAT1* | F: GCTCTGGTACAGCCTGTGTTGT  R: TGCCAGCTTAGGACCATTCTCC |
| Human | *PHGDH* | F: CTTACCAGTGCCTTCTCTCCAC  R: GCTTAGGCAGTTCCCAGCATTC |
| Human | *EIF2AK3* | F: ACGATGAGACAGAGTTGCGAC  R: ATCCAAGGCAGCAATTCTCCC |
| Human | *S100A11* | F: ATGGCAAAAATCTCCAGCCCT  R: TGTGAAGGCAGCTAGTTCTGTA |
| Human | *CEP55* | F: AGTAAGTGGGGATCGAAGCCT  R: CTCAAGGACTCGAATTTTCTCCA |
| Human | *SULF2* | F: TTGCCGTGTACCTCAATAGCA  R: TTCCGACACAGCGTGTAGTTA |
| Human | *STC2* | F: GCGTGCAGGTTCAGTGTGA  R: GGCCAGTCTCCCTACTGCT |
| Human | *CCND1* | F: CCCTCGGTGTCCTACTTCAA  R: GTGTTCAATGAAATCGTGCG |
