# Supplementary material for: VRK1 promotes epithelial-mesenchymal transition in hepatocellular carcinoma mediated by SNAI1 via phosphorylating CHD1L
Source: Cell Death Dis. 2025 Apr 15;16(1):302. doi: 10.1038/s41419-025-07641-w (PMC12000354; doi:10.1038/s41419-025-07641-w)

Fig 2A

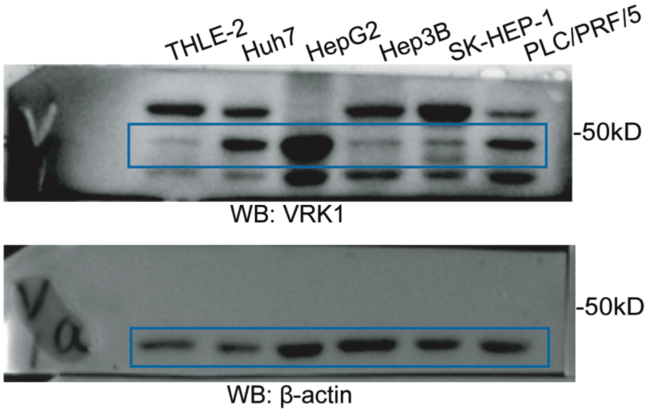
THLE-2
Huh7
HepG2
Hep3B
SK-HEP-1
PLC/PRF/5
-50kD
WB: VRK1
-50kD
WB: β-actin

Fig 2C

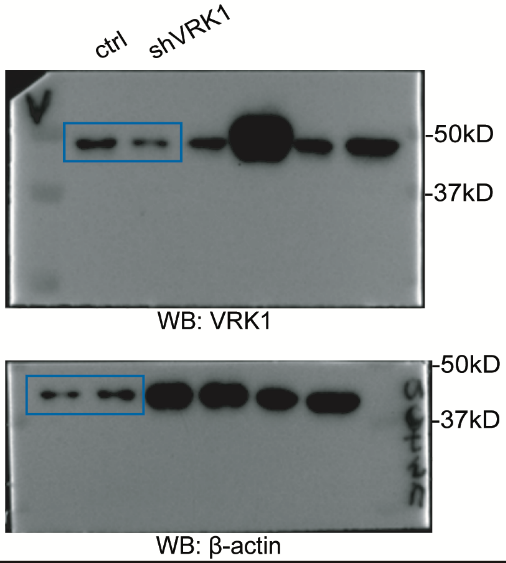
ctrl
shVRK1
-50kD
-37kD
WB: VRK1
-50kD
-37kD
WB: β-actin

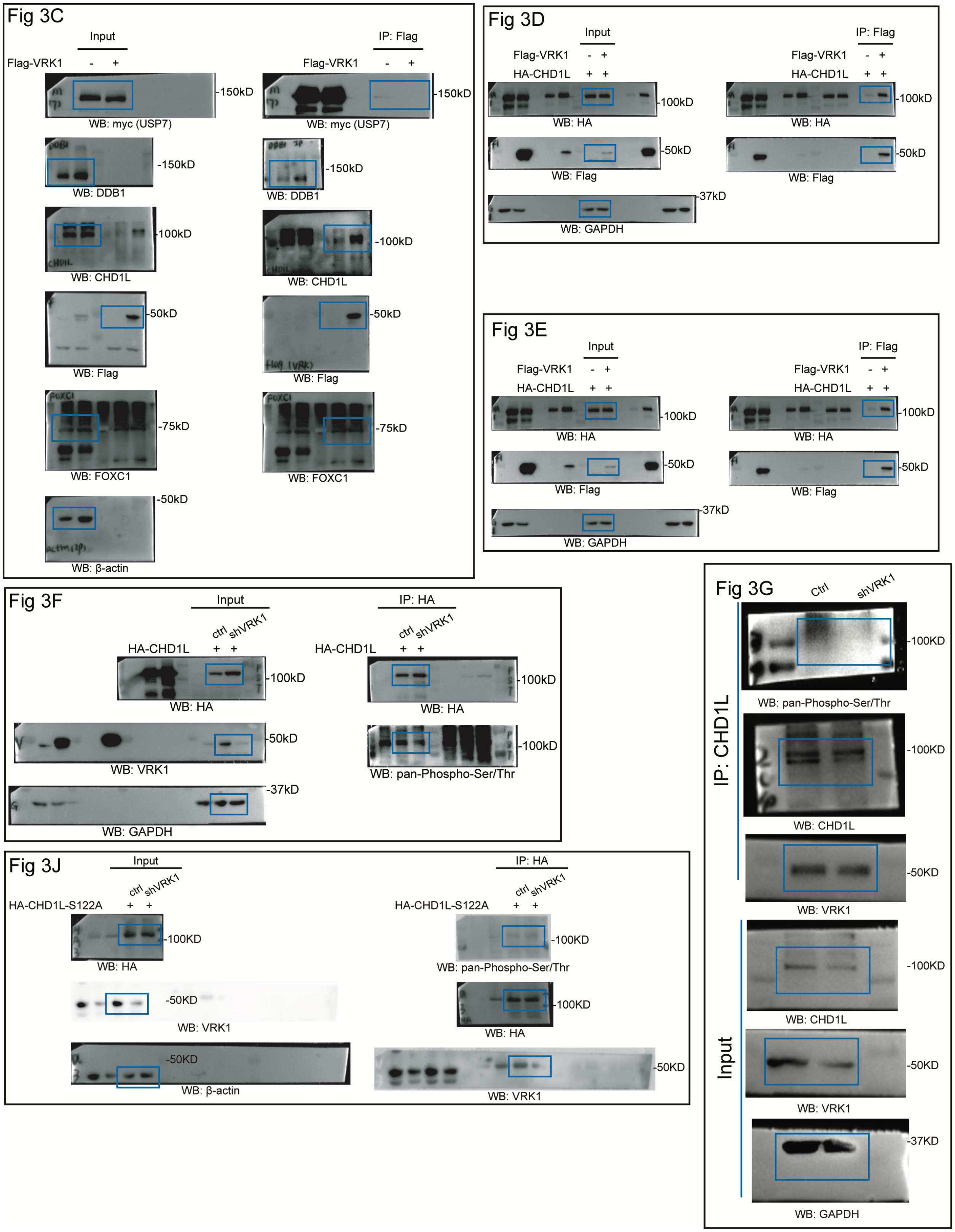
Fig 3C
Input
IP: Flag
Flag-VRK1 - +
Flag-VRK1 - +
-150kD
WB: myc (USP7)
-150kD
WB: myc (USP7)
-150kD
WB: DDB1
-150kD
WB: DDB1
-100kD
WB: CHD1L
-100kD
WB: CHD1L
-50kD
WB: Flag
-50kD
WB: Flag
-75kD
WB: FOXC1
-75kD
WB: FOXC1
-50kD
WB: β-actin
Fig 3D
Input
IP: Flag
Flag-VRK1 - +
HA-CHD1L + +
Flag-VRK1 - +
HA-CHD1L + +
-100kD
WB: HA
-100kD
WB: HA
-50kD
WB: Flag
-50kD
WB: Flag
-37kD
WB: GAPDH
Fig 3E
Input
IP: Flag
Flag-VRK1 - +
HA-CHD1L + +
Flag-VRK1 - +
HA-CHD1L + +
-100kD
WB: HA
-100kD
WB: HA
-50kD
WB: Flag
-50kD
WB: Flag
-37kD
WB: GAPDH
Fig 3F
Input
IP: HA
ctrl shVRK1
HA-CHD1L + +
ctrl shVRK1
HA-CHD1L + +
-100kD
WB: HA
-100kD
WB: HA
-50kD
WB: VRK1
-100kD
WB: pan-Phospho-Ser/Thr
-37kD
WB: GAPDH
Fig 3G
Ctrl
shVRK1
IP: CHD1L
-100KD
WB: pan-Phospho-Ser/Thr
-100KD
WB: CHD1L
-50KD
WB: VRK1
Input
-100KD
WB: CHD1L
-50KD
WB: VRK1
-37KD
WB: GAPDH
Fig 3J
Input
IP: HA
ctrl shVRK1
HA-CHD1L-S122A + +
ctrl shVRK1
HA-CHD1L-S122A + +
-100KD
WB: HA
-100KD
WB: pan-Phospho-Ser/Thr
-50KD
WB: VRK1
-100KD
WB: HA
-50KD
WB: β-actin
-50KD
WB: VRK1

Fig 4H

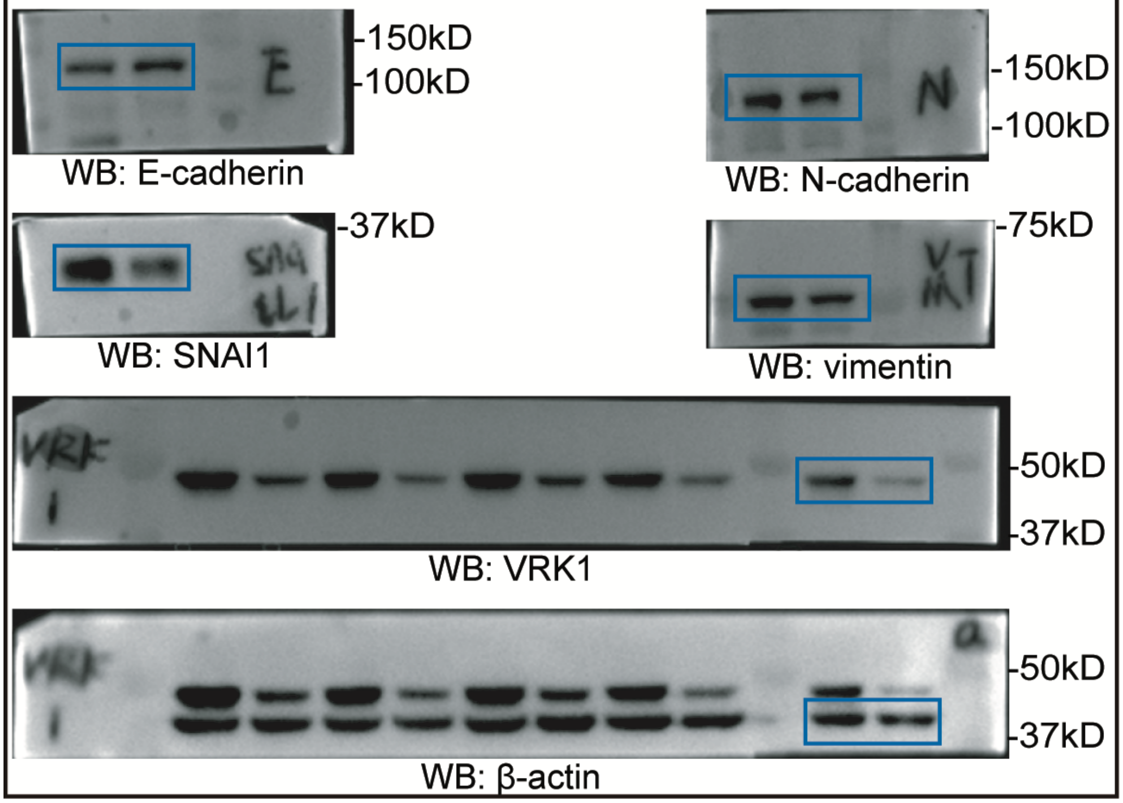
-150kD
-100kD
WB: E-cadherin
-150kD
-100kD
WB: N-cadherin
-37kD
WB: SNAI1
-75kD
WB: vimentin
-50kD
-37kD
WB: VRK1
-50kD
-37kD
WB: β-actin

Fig 5H

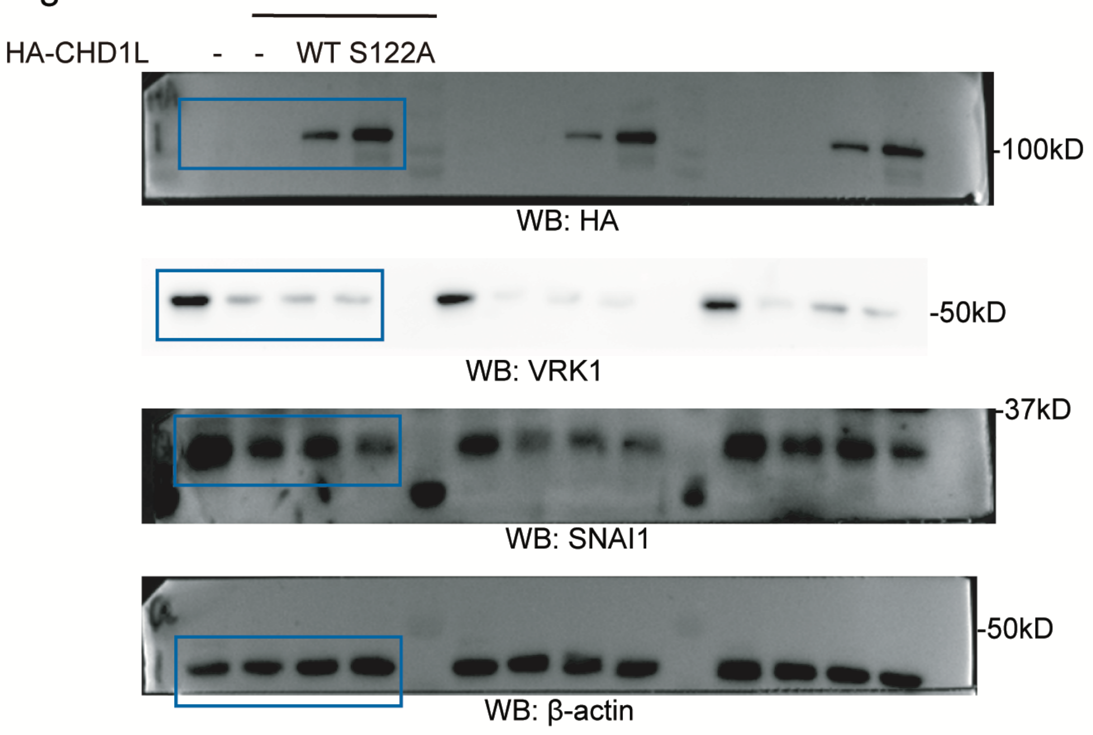
ctrl
shVRK1
HA-CHD1L
-
-
WT S122A
-100kD
WB: HA
-50kD
WB: VRK1
-37kD
WB: SNAI1
-50kD
WB: β-actin

Fig 6F

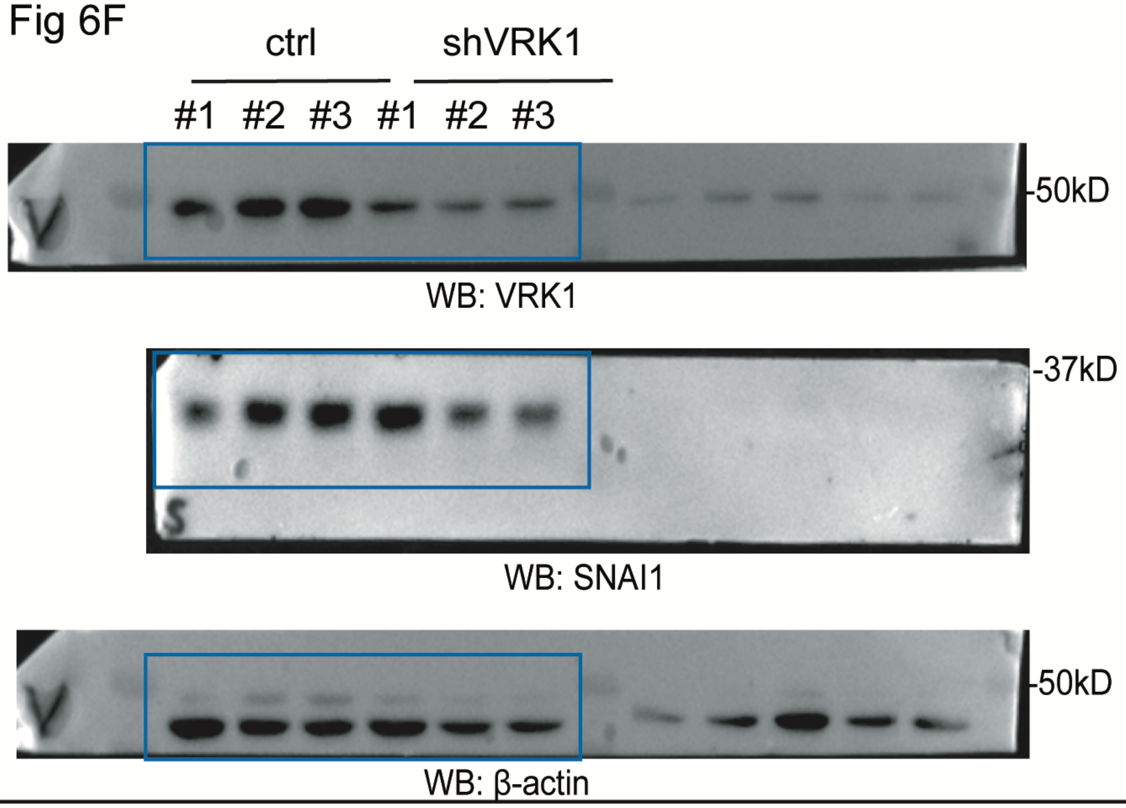
ctrl
shVRK1
#1 #2 #3 #1 #2 #3
-50kD
WB: VRK1
-37kD
WB: SNAI1
-50kD
WB: β-actin

Fig 6G

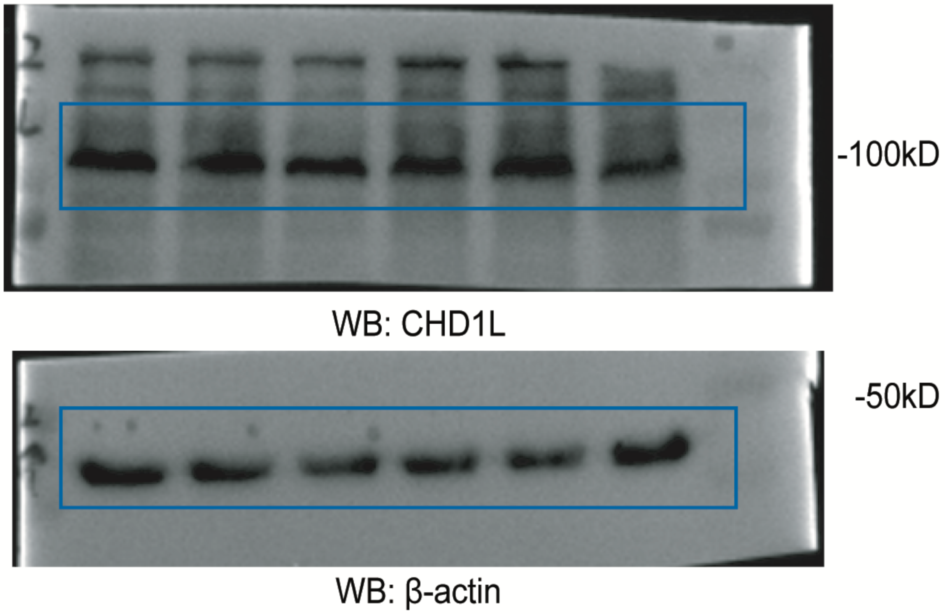
-100kD
WB: CHD1L
-50kD
WB: β-actin

Supplementary Figure1

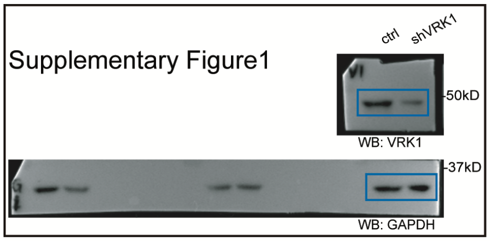

Supplementary Figure2C

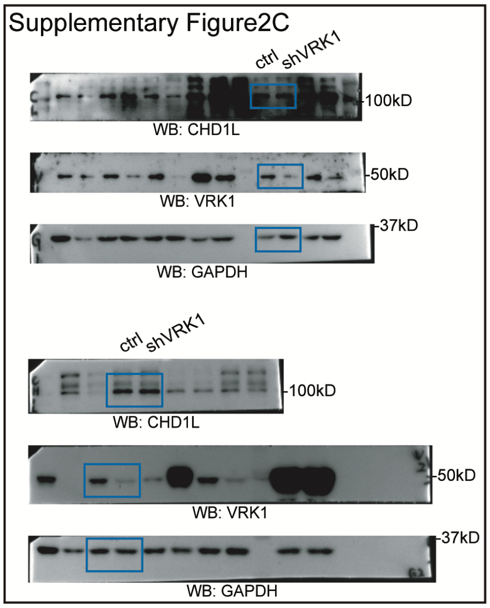

Supplementary Figure2

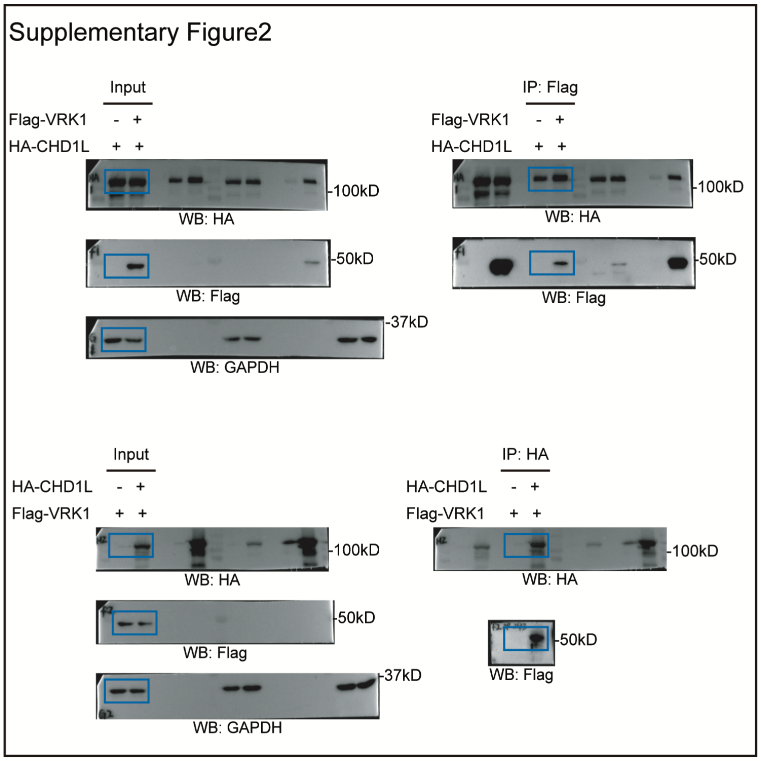

Supplementary Figure3

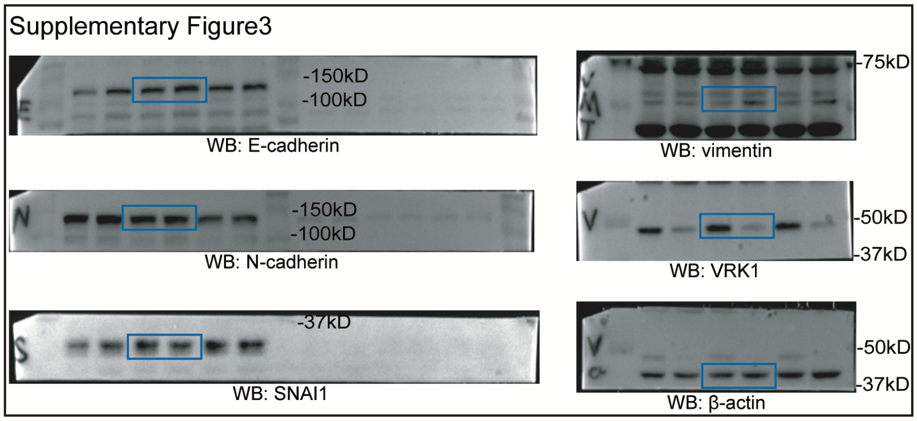

Supplementary Figure 4

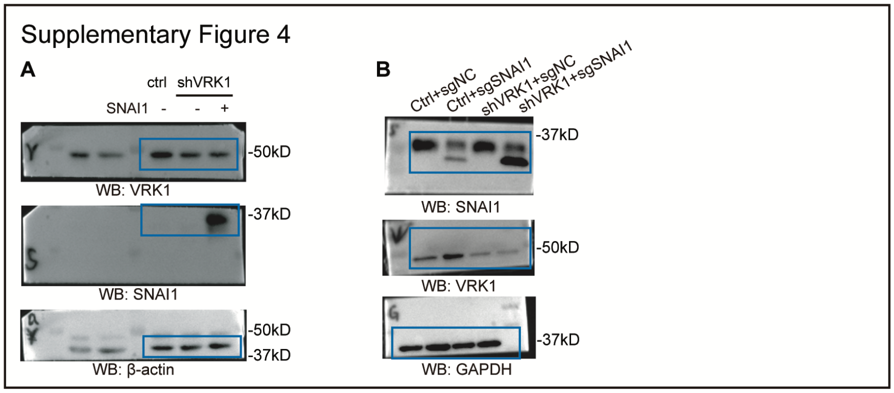

Supplement: Supplementary file 7 — Western Blot Original Data [file 41419_2025_7641_MOESM7_ESM.pdf]
